# HIV induces production of IL-18 from intestinal epithelial cells that increases intestinal permeability and microbial translocation

**DOI:** 10.1371/journal.pone.0194185

**Published:** 2018-03-30

**Authors:** Ossama Allam, Suzanne Samarani, Vikram Mehraj, Mohammad-Ali Jenabian, Cecile Tremblay, Jean-Pierre Routy, Devendra Amre, Ali Ahmad

**Affiliations:** 1 Laboratory of Innate Immunity, CHU Ste-Justine Research Center/Department of Microbiology, Infectious Diseases & Immunology, University of Montreal, Montreal, Québec, Canada; 2 Division of Hematology & Chronic Viral Illness Service, McGill University, Montreal, Québec, Canada; 3 Department of Biological Sciences, UQAM, Montreal, Québec, Canada; 4 CHUM/ Department of Microbiology, Infectious Diseases & Immunology, University of Montreal, Montreal, Québec, Canada; 5 CHU Ste-Justine Research Center/Department of Pediatrics, University of Montreal, Montreal, Québec, Canada; Karolinska Institutet Department of Medicine Solna, SWEDEN

## Abstract

Interleukin-18 (IL-18) is a pleiotropic cytokine of the IL-1 family with multiple context dependent functions. We and others have shown that HIV infection is accompanied by increased circulating levels of IL-18 along with decreased levels of its antagonist, Interleukin-18 Binding Protein (IL-18BP). The infection is also accompanied by intestinal inflammation and decreased intestinal integrity as measured by intestinal permeability, regeneration and repair. However, little is known concerning the relation between high level of IL-18 associated with the viral infection and intestinal permeability. Here we demonstrate that HIV treatment increases production of IL-18 and decreases that of IL-18BP production in human intestinal epithelial cell (IEC) lines. IL-18 causes apoptosis of the IEC by activating caspase-1 and caspase-3. It induces epithelial barrier hyperpermeability by decreasing and disrupting both tight and adherens junction proteins, occludin, claudin 2 and beta-catenin. Disorganization of F-actin was also observed in the IEC that were exposed to the cytokine. Moreover IL-18 decreases transepithelial electrical resistance (TEER) in Caco-2 and increases permeability in HT29 monolayers. The cells’ treatment with IL-18 causes an increase in the expression of phosphorylated myosin II regulatory light-chain (p-MLC) and myosin light-chain kinase (MLCK), and a decrease in phosphorylated Signal Transducer and Activator of Transcription (p-STAT)-5. This increase in p-MLC is suppressed by a Rho-kinase (ROCK)-specific inhibitor. Interestingly, the levels of the cytokine correlate with those of LPS in the circulation in three different categories of HIV infected patients (HAART-naïve and HAART-treated HIV-infected individuals, and Elite controls) as well as in healthy controls. Collectively, these results suggest that the HIV-induced IL-18 plays a role in increased intestinal permeability and microbial translocation observed in HIV-infected individuals.

## Introduction

Interleukin 18 (IL-18), originally named as the Interferon-γ (IFN-γ)-inducing factor, is a pro-inflammatory cytokine that belongs to the IL-1 family [1]. Like IL-1β, the prototype member of the family, it is produced as an inactive 33 kD precursor, which is processed by caspase-1 into mature and biologically active 17 kD form. The caspase-1 itself needs activation via assembly of an inflammasome. In the circulation, the mature (m) IL-18 is bound and inactivated by IL-18 binding protein (IL-18BP), which is produced as a negative feed-back mechanism in response to increased concentration of the cytokine. IL-18BP protects body from tissue destructive effects of the cytokine [2,3]. IL-18 is produced in the body from a wide variety of cells including epithelial cells, macrophages, dendritic cells, keratinocytes, adrenal cortex and platelets [3–5]. It can perform multiple context-dependent biological functions. It induces IFN-γ from NK and T cells in the presence of IL-12, and drives TH1-like immune responses. However, the cytokine promotes TH2 type responses in the absence of IL-12 by inducing IL-4 from eosinophils, basophils and naïve T cells [6,7]. It also induces FasL expression on NK and T cells [8]. The cytokine has also been shown to induce death in a variety of human cells (eg, vascular and cardiac endothelial cells [9]. Its role in the pathophysiology of gut is complex. Steady levels of the cytokine are protective as they control outgrowth of colitogenic bacteria and maintain integrity of the intestinal epithelium. Consequently, IL-18 KO mice are more susceptible to the dextran sulfate sodium (DSS)-induced colitis [10,11]. However, overwhelming evidence suggests that increased concentrations of the cytokine play an important role in intestinal inflammation. Recent studies, in this regard, have shown that intestinal epithelial cell (IEC)-produced IL-18 controls intestinal barrier function and promotes DSS-induced colitis by inhibiting differentiation, and promoting depletion, of goblet cells [12]. The cytokine concentrations are increased in the circulation of Inflammatory Bowel Disease (IBD) patients and they correlate with severity of mucosal inflammation [13]. Furthermore, in vivo neutralization of IL-18 with neutralizing antibodies, IL-18BP or anti-sense oligos ameliorates inflammation in murine models of colitis [14–16].

Previous studies from our [17,18] and other laboratories [19,20] have shown that IL-18 concentrations are increased in the circulation of HIV-infected individuals. The cytokine is known to drive HIV replication in both T cells and macrophages [21,22]. Its concentrations in the circulation were shown to correlate positively with cell free viral DNA and negatively with CD4+ T cell counts [20]. Interestingly, while concentrations of the cytokine increase, those of its antagonist (IL-18BP) decrease or are not correspondingly increased resulting in increased biological activities of the cytokine; reviewed in [2]. Given that IEC represent an important source of IL-18 and HIV infection leads to a loss in gut barrier function and enhanced microbial translocation [3,23,24], we sought to investigate whether the virus has any effect on the expression and activation of this cytokine from these cell types. The issue gains more significance in view of the fact that gastro-intestinal tract (GIT)-associated lymphoid tissue is the primary site where HIV replicates and causes death of CD4+ T cells [25,26]. The localized viral replication compromises intestinal barrier function, which is normally maintained primarily by the Tight Junction (TJ) proteins comprising claudins, and occludin, etc; reviewed in [27]. These proteins form a tight gasket between adjacent intestinal epithelial cells and regulate paracellular movement of solutes. The function of TJ is supported by another complex located underneath it, called Adherens Junctions (AJ), which comprises E-cadherins, beta-catenin and ZO-1 [28]. It has been well-documented that the intestinal barrier function is compromised in HIV-infected individuals early in the course of the infection. The intestines in these individuals are five-fold more permeable compared to their healthy counterparts resulting in increased translocation of microbial products (e.g., LPS) in the circulation [29]. The increased translocation of microbial products is widely believed to cause increased systemic activation of the immune system in HIV-infected individuals. Although highly active anti-retroviral therapy (HAART) suppresses HIV replication to undetectable levels, low level replication of the residual virus results in chronic low-grade inflammation, which contributes towards many non-AIDS-associated clinical conditions (such as frailty, immune senescence, accelerated aging, metabolic syndrome, cancer and lipodystrophy) in HIV-infected individuals receiving HAART [30,31].

Using human IEC lines, we show here that HIV induces IL-18 production but inhibits that of its antagonist in these cell types. The cytokine increases intestinal permeability in the IEC monolayers and causes cell death. The cytokine may represent an important soluble mediator that promotes AIDS progression by increasing intestinal permeability and cell death.

## Materials and methods

### Reagents

Different reagents used in this study were purchased as follows: recombinant human IL-18 (rh IL-18) from MBL (Code #B001-5), serum-free medium from Gibco (catalogue # 120-550-91), recombinant HIV-1 protein Tat (Clade B) from ProSpec (Protein-Specialists; catalog # HIV-129), Lipopolysaccharide (LPS) from Sigma-Aldrich (catalog # L4005), the Rho kinase (ROCK) inhibitor GSK 269962 from Santa Cruz Biotechnology (catalog # sc-363279), the cell permeable irreversible caspase-1 inhibitor (Z-YVAD-FMK) from Santa Cruz (catalog #sc 3071) and cell permeable caspase-3 inhibitor (Z-DQMD-FMK) from Tocris (catalog # 2168). In some experiments, Tat was used after its pre-incubation with Tat-neutralizing polyclonal rabbit antibodies from Sigma-Aldrich (catalog # HPA029316) or with rabbit IgG from non-immunized animals (EMD Millipore (12–370).

### Intestinal epithelial cell (IEC) culture

Human colonic epithelial cells Caco2 (ATCC^®^ HTB37^™^) were cultured in monolayers in Eagle’s Minimum Essential Medium (EMEM; Wisent Bioproducts catalog # 320-012-cl) with 15% heat inactivated FBS, 2mM L-glutamine and antibiotics in 37°C, 5% CO_2_ and 90% relative humidity. Caco2 cells were used in experiments between passages 16–24. Human colon adenocarcinoma grade II cell line HT-29 (ATCC HTB-38) was cultured in monolayers with McCoy’s medium with 10% FBS and antibiotic. HT-29 cells were used between passages 9–19. The culture media were changed every three days and the cells were sub cultured when the monolayers reached 80–90% confluence. The treatments of the cells were performed in the serum-free medium. For sub-culturing, the monolayers were treated with trypsin-EDTA from (Sigma-Aldrich) or with Versene 1X from Life Technologies (Ref # 15040–066) for apoptosis assay experiments.

### Western blots

Expression of different proteins was determined in the intestinal epithelial cell line HT-29. The cells were suspended in the lysis buffer containing Tris-HCl (pH 6.8), 2% sodium dodecyl sulfate (SDS), and cocktail of protease inhibitors (Sigma-Aldrich, Catalog number P8340) at 1μl/0.1ml of lysates. The cells were lysed by sonication for 20 seconds. The lysates were clarified by centrifugation for 15 min at 14,000 *g* at 4°C. The lysate proteins were resolved on 10% or 12% SDS-polyacrylamide gel electrophoresis (PAGE) under reducing conditions as described [8]. The resolved proteins were electroblotted onto nylon membranes (Cat # IPVH00010; Immobilon-P; from EMD Millipore). The unbound sites on the membranes were blocked with casein (Vector Laboratories Catalog No SP-5020, concentration 1:10). The primary antibodies used for detecting bands for Western blots were: rabbit anti-IL-18 from MBL (catalogue # PM014); goat anti-IL-18BP from R&D Systems (catalogue #AF 119); rabbit anti human Myosin Light Chain (MLC)-2 (catalogue # 3672), mouse monoclonal anti-phospho-MLC-2 (Ser19) (catalogue # 3675), rabbit anti-Stat-5 (catalogue # 9363), rabbit anti-phospho-Stat5 (Tyro 694) (catalogue # 9351), rabbit anti-human caspase-1 (catalogue # 2225) and rabbit anti-human caspase-3 antibody (Catalog #9662) from Cell Signaling Technology; mouse anti-human GAPDH (G-8795) and rabbit (IgG) anti-human β-actin (A-5060) were from Sigma-Aldrich; rabbit polyclonal anti-human occludin from Invitrogen (cat#40–4700) and rabbit (IgG) anti-human claudin-2 from Santa Cruz Biotechnology (cat#133464), and mouse anti-myosin light chain kinase (MLCK) monoclonal antibody from Abcam (ab55475). Biotinylated secondary antibodies included in the Vectastatin kits from Vector Laboratories were used following the kit instructions. Each Western blot was repeated at least thrice. Band densities were determined by using Image J.

### Immunofluorescence microscopy

Caco2 and HT-29 cells were seeded on round glass coverslips in six-well plates for four days. On the fourth day, the cells (confluence 60–80%) were treated with 10 ng/ml IL-18 and Tat protein 100 ng/ml for 24 hours and were used for Immunofluorescence staining. The cells were permeabilized and fixed by incubation with methanol 50% and PFA 4% for 10 min on ice. After washing the coverslips three times by PBS containing 2% FBS for 5 minutes, the cells were incubated with 10% rabbit serum and 10% FBS for 1 hour on ice. The following antibodies were used in 4°C overnight as first antibodies: polyclonal rabbit anti-β-catenin (catalog # 9562) and the antibodies for occludin and caludin-2 were the same as described in the section on Western blots. After 24 hours’ incubation with the first antibody at 4 °C, the cells were washed three times with PBS. Goat F(ab’)2 FITC-conjugated anti-rabbit from eBioscience (Ref # 11-4839-81) was used as the secondary antibody. The secondary Ab was incubated with cells for one h on ice. Actin filaments were stained by Tetramethylrhodamine B isothiocyanate-conjugated Phalloidin from Sigma Aldrich (Catalog Number P1951) by incubation with cells for one hour on ice. Nuclei were stained with 4',6-diamidino-2-phenylindole (DAPI) (at 1:5000 dilution; Biolegend; cat#422801). The stained cells were examined by the Eclipse e-800 microscope, Nikon and images were taken at 400 magnification.

### Virus preparation and treatment

Infectious proviral DNA for a T-tropic viral strain NL4.3 and a dual tropic viral strain 86.9 was amplified from their respective plasmids (pNL4.3 and p89.6, respectively) using a commercial kit (Midiprep kit, Qiagen, Ontario, CA). Viral preparations were made by transfecting HEK293-T cells (an adenovirus-transformed human embryonic kidney cell line expressing the SV-40 large T antigen; ATCC; CRL-11268) with 1.00 ug proviral DNA using a polyethylenimine (catalog # 408712 from Sigma-Aldrich) in an in-house developed protocol. The transfected cells were incubated in the medium (RPMI) at 37°C in humidified 5% CO_2_ atmosphere. After 24 hours, culture supernatants were collected, clarified by centrifuged at (14,000 *g*). The supernatants were titrated for p24 contents using a commercial ELISA kit from ABL (Rockville, USA). The supernatants were aliquoted and stored at -80°C. The supernatants from mock-transfected HEK293-T cells were used as mock viral preparations.

HT-29 cells were incubated with NL4.3 or p89.6 HIV-1 virus strains (at 1 moi) for 24 hours as described before [18]. After incubation, the cells were washed 3 times with PBS and lysed for Western blot. The levels of IL-18 and IL-18BP in the supernatants were measured by ELISA after deactivating the virus by 1% Titron 100X (Sigma-Aldrich) for one hour.

### The transendothelial electrical resistance (TEER) in Caco2 cells

Caco2 (2x10^4^ cells) were seeded on the Transwell filter insert or on a gelatin-coated 3-μm pore size Boyden chamber from BD Biosciences. DMEM culture medium was changed every day. On the experiment day, Caco2 cells were treated with 10 ng/ml of IL-18 and/or with 100 ng/ml of HIVs’ recombinant Clade-B Tat protein (from ProSpec; catalog # HIV-129). For each treatment, three replicates were used. Electrical resistance was measured over 24 hours in non-treated Caco2 cells and the both treated cells as previously described [32]. TEER was expressed as Ohm (resistance) x cm^2^ (surface area of monolayer).

### Measurement of Tight Junction permeability with Lucifer yellow in HT-29 cells

HT-29 cells were seeded on 24-well plate Boyden chamber inserts (Becton Dickinson Labware; catalog # 353096) and placed in 24-well plates (Becton Dickinson Labware; cat# 353504). When the microcultures reached 90% confluence, recombinant human (rh) IL-18 10 ng/ml, neutralized rhIL-18 10 ng/ml (pre-mixed with IL-18 neutralizing antibodies) or TGF-β1 (10 ng/ml) was added to the microcultures in the apical or basal positions. After 16 h of incubation, cells were washed 3 times with PBS. The medium in the lower chambers was replaced with 200 ul of PBS. Thereafter, 100 nMol of the fluorescence tracer Lucifer Yellow CH dilithium salt (LY; 0.45 kDa; Sigma-Aldrich) was added to the upper chambers. After 30 minutes, upper chambers were removed, and PBS from the lower chambers was collected and transferred to a 96-well ELISA plate to measure fluorescence intensity at 428 nm excitation and 530 nm emission wavelengths.

### Apoptosis assay

The percentage of apoptotic cells were measured by using Annexin V-FITC Apoptosis Detection Kit from eBioscience (catalog # BMS500FI). Briefly, HT29 cells were collected by using Versene Solution from Life Technology. Cells were stained with FITC-Annexin V for 15 minutes, washed and stained with propidium iodide (PI) and apoptotic cells were quantified by flow cytometry using FACSCalibur (BD Biosciences as described earlier [33].

### Measurement of LPS, IL-18 and IL-18BP

Levels of lipopolysaccharide (LPS), IL-18 and IL-18BP in HIV infected individuals and healthy volunteers’ plasma samples were measured using commercial ELISA kits. Plasma samples from 15 treatment-naïve and 15 HAART-treated individuals with chronic HIV infection, 9 Elite controllers and 17 HIV-seronegative healthy donors were used for these determinations. Clinical data for all the study participants are shown in Table 1. Free IL-18 was measured by using the Human IL-18 Instant ELISA kit from eBioscinece (REF# BMS267 INST). The lower detection limit of the kit was 78 pg/ml. The plasma LPS levels were determined by the Human LPS ELISA Kit from MyBioSource (Catalog # MBS702450). The detection range of the kit was 6.25 pg/ml -400 pg/m. IL-18BP isoform “a” was determined in cell culture supernatant by a ELISA kit from R&D Systems (catalog #DY119) with the detection range of 93.8–6,000 pg/mL.

**Table 1:** Clinical parameters of study participants.

Category	Number of patients	Mean CD4+ T cell count	Mean CD8+ T cell count	Viral load
HAART-Naïve	15	551	744	3.5895
HAART-Positive	15	486	798	<1.70
Elite	9	826	502	<1.60
Healthy	17	792	367	NA

CD4^+^ and CD8^+^ T cell counts are given per mm^3^. Viral loads are in RNA copies in log_10_ per ml. NA means not applicable.

### Ethical statement

All research involving human participants was approved by the CER (Committee for Ethics in Research) of the Centre hospitalier universitaire (CHU) Sainte-Justine, Montreal. All clinical investigations were conducted according to the principles expressed in the Declaration of Helsinki. Informed written consent was obtained from the participants. The data were analyzed anonymously and reported.

### Statistical analysis

Group means were compared using one way non-parametric ANOVA (Kruskal-Wallis test) with pairwise Mann-Whitney’s post-hoc tests. Correlation between two parameters was determined by Pearson correlation. The software used for statistical analyses was the Graphad PRISM5, San Diego, CA. The p values ≤0.05 were considered as statistically significant.

## Results

### Effects of HIV and the viral protein Tat on IL-18 and IL-18BP production from HT-29 cells

**Fig 1:**
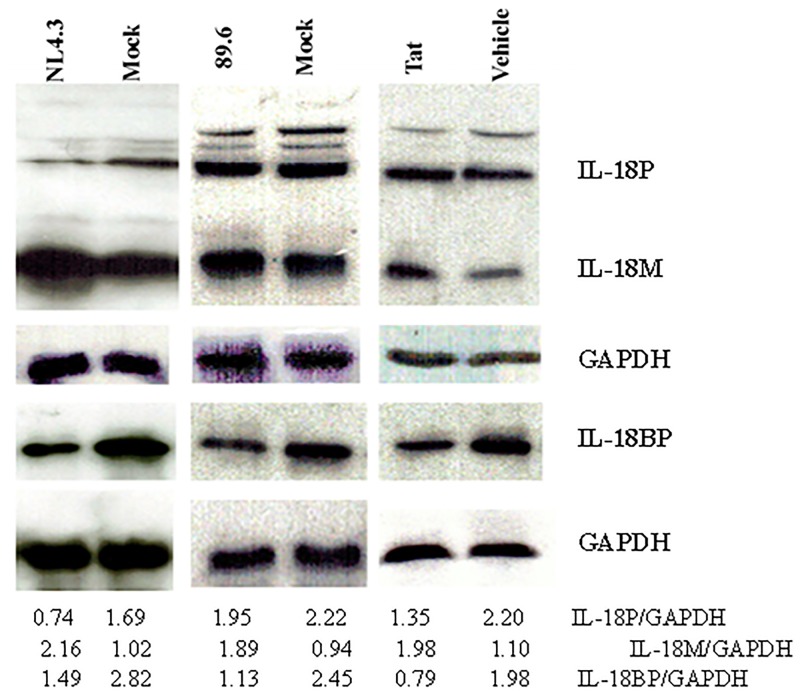
HIV treatment increases IL-18 but decreases IL-18BP in IEC. HT-29 monolayers (~ one million cells) were infected with 10^6^ infectious units of NL4.3, 89.6, or treated with Tat (100 ng/ml). After 24 hours, precursor IL-18 (IL-18P), mature IL-18 (IL-18M) and IL-18BP were detected in the cell lysates (25 ug of the lysate proteins) by Western blots. The Figure shows a representative of several experiments. GAPDH was detected as a control for protein loading. Note that the viral as well as Tat treatment increases expression of mIL-18 and decreases that of IL-BP. The ratios of band densities for each protein to the control are shown below the blot.

**Fig 2:**
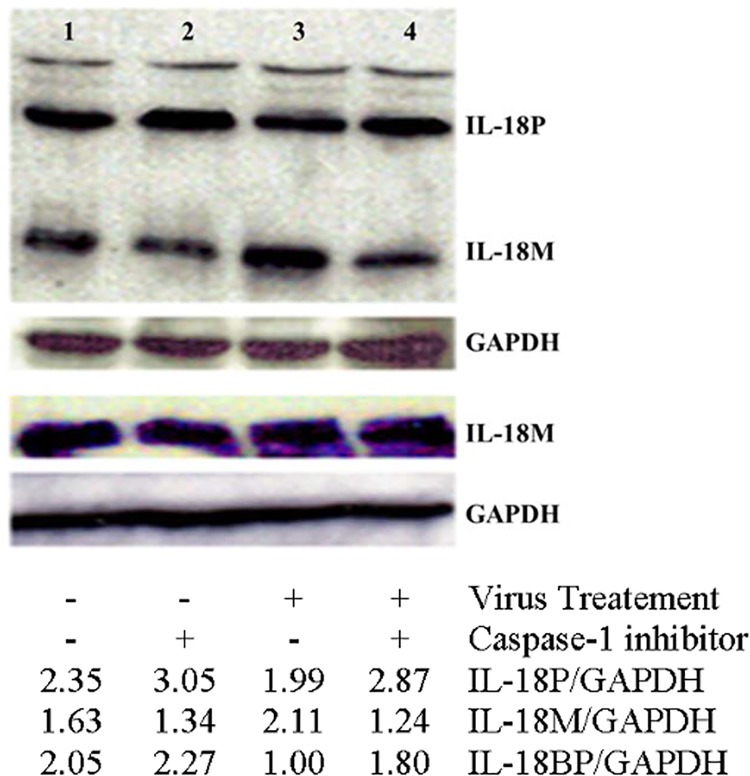
The effect of caspase-1 inhibitor on HIV-induced changes in IL-18 and IL-18BP in IEC. HT-29 monolayers were treated with NL4.3 at moi 1 with and without prior treatment with caspase-1 inhibitor (Z-YVAD-FMK; 50 uM). The inhibitor was added 5–10 minutes prior to the treatment with viral preparations. IL-18, IL-18BP and GAPDH were detected and quantified as described in the legend to Fig 1.

The IEC line HT-29, like other intestinal epithelial cells, can produce both IL-18 and its antagonist, IL-18BP, upon proper stimuli [23,34]. Although, the cells are not susceptible to HIV infection, they can interact with the virus through a variety of their surface-expressed molecules such as galactosylceramide, heparan sulfate proteoglycans, Clec2, CXCR4, and CCR5 etc., [26,35–37]. Therefore, we used these cells to investigate the potential effects of the virus and the viral transactivator Tat on the production of these two soluble mediators. Tat is an important HIV protein that contributes towards pathogenesis of HIV infection by multiple mechanisms [38,39]. The cells were incubated separately with viral preparations made from a CXCR4-troic (NL4.3) and a dual-tropic (89.6) viral strain and with their respective mock preparations. Recombinant Tat or vehicle was also used in some experiments. After 24 hours, the cells and the culture supernatants were collected. We detected precursor IL-18, mature IL-18 and IL-18BP in the cell lysates by Western blots using specific antibodies. As shown in Fig 1, the viral and Tat treatment each caused increases in the levels of mIL18 but not in those of pIL-18. In fact, the treatments caused a decrease in the expression of pIL-18 compared to their respective controls. Furthermore, the treatments also caused a decrease in the levels of IL-18BP in the cells as determined by Western blots (Fig 1). A decrease in pIL-18 was probably due to its more processing into its mature form. An increase in mIL-18 suggested increased processing of the pIL-18. As caspase-1 is known to process intracellular pIL-18 into mIL-18, we sought to determine the effect of a caspase-1 inhibitor on the HIV-induced increase in mIL-18. As shown in Fig 2, prior treatment of the virus-treated cells with a cell permeable caspase-1 inhibitor prevented increase in mIL-18 inside cells but had no effect on IL-18BP levels.

**Fig 3:**
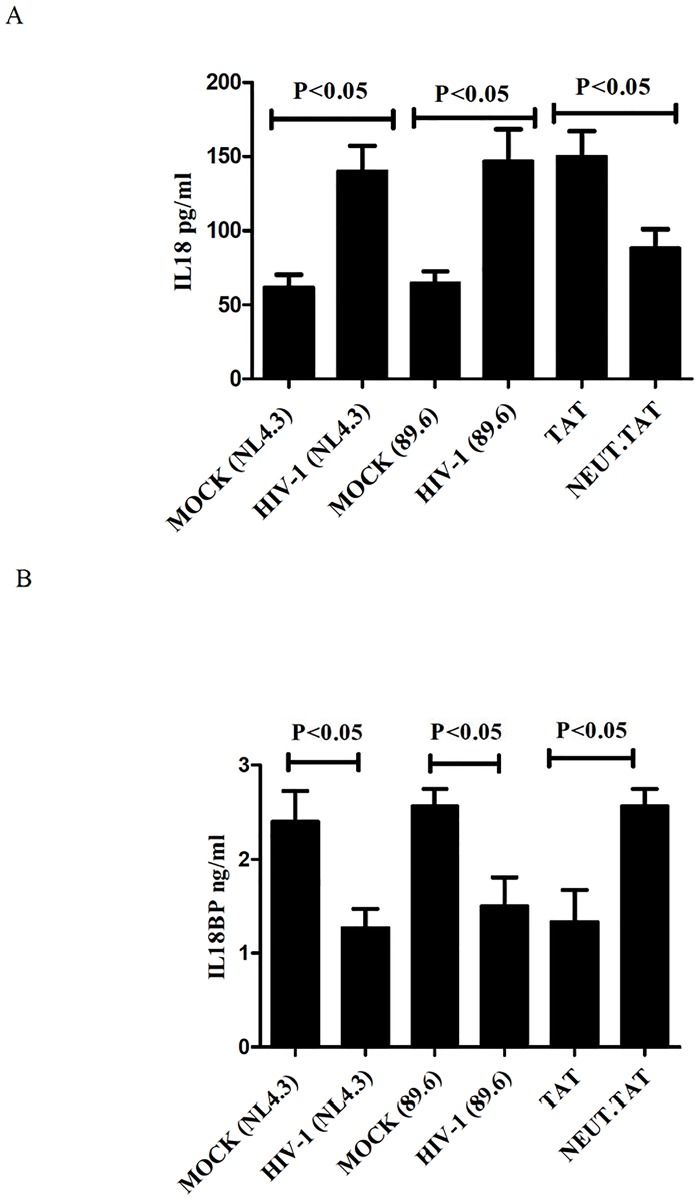
The effects of HIV and Tat treatments on the secretion of IL-18 and IL-18BP from IEC. The panels A and B show mean concentrations of IL-18 and IL-18BP, respectively, in the culture supernatants. The vertical line on each bar denotes standard deviation. The Tat used in these experiments was pre-treated with Tat-neutralizing antibodies (Neut Tat) or with control antibodies (Tat).

Next we determined the effects of HIV and Tat treatments on the secretion of mIL-18 and IL-18BP. We measured their concentrations in the culture supernatants of the virus-treated and Tat-treated HT-29 cells. Results from three independent experiments (each with three replicates) are shown in Fig 3 panel A. The viral and the Tat treatments all increased mIL-18 concentrations in the culture supernatants significantly (p<0.01). On the other hand, these treatments decreased the concentrations of IL-18BP in the culture supernatants (Fig 3 panel B). As expected, in these experiments, treatment of Tat with Tat-neutralizing antibodies abrogated the Tat-exerted effects on the secretion of mIL-18 indicating that the effect is specific to Tat, and not due to any contaminants present in this preparation.

### IL-18 induces death in HT-29 cells dependent upon dose and time

**Fig 4:**
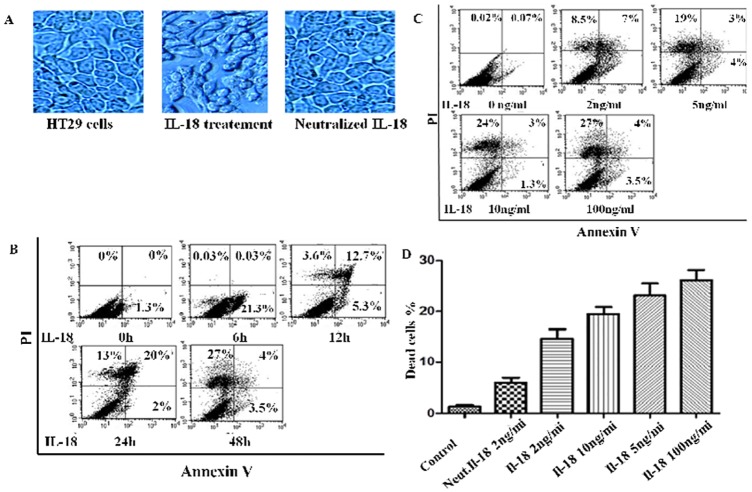
IL-18 induces death in HT-29 cells. Light microscope images (x100) of the cells taken on day 5 after treatment with 100 ng per ml of IL-18 (**A**). Note that prior neutralization of the cytokine with an IL-18 neutralizing monoclonal antibody (10 ug per ml) inhibited the deleterious effects of the cytokine. **B**. Staining of the IL-18-treated cells for FITC-conjugated Annexin V and PI at different time points. Note earlier staining of the cells with FITC-Annexin V followed by uptake of PI. The temporal pattern of the two stains is characteristic of apoptosis. **C**. The cytokine induces death in a dose dependent manner. All these experiments were performed at least three times and results from a representative experiment are shown in A, B and C. The data from three independent experiments (mean ± SE) is shown in **D**. Each dose induced significantly more cell death compared with the untreated cells; the neutralization of IL-18 inhibits the death significantly (p<0.05).

We and others have shown that IL-18 can induce death in certain cell types [8,9,340], therefore we sought to determine the effects of recombinant human IL-18 on these IEC. The cytokine was pre-treated with an IL-18 neutralizing monoclonal antibody from MBL (cat # D044-3) or with mouse IgG1 obtained from non-immunized animals (Abcam; # ab91353). The cytokine induced morphological changes in HT-29 cells grown in monolayers. The cells became progressively rounded, detached and underwent death. Fig 4 panel A shows cell death on day 5 after treatment of the HT-29 monolayers with IL-18 (100 ng per ml). As expected, prior incubation of the cytokine with an IL-18 neutralizing monoclonal antibody abrogated the death inducing effects of the cytokine. These data suggest that the effects of the cytokine were specific and were not due to any contaminant present in its preparation. In order to determine the mode of cell death, the cells were treated with the cytokine, and analyzed for their staining with FITC-conjugated Annexin V and PI at different time points. The cells became positive for Annexin V (a marker of early apoptosis) 6 hours after the treatment. Thereafter at 12 hours, they became positive for Annexin V and PI (advanced apoptosis), and finally at 24 hours they became positive for PI (late apoptosis with loss of membrane integrity; Fig 4 panel B). This pattern of temporal positivity for the two stains is typical of apoptosis. We measured cell death (PI positive cells) at different doses of the cytokine. As shown in Fig 4 (panels C and D), the cytokine induced cell death in a dose dependent manner.

### IL-18-induced cell death is partially inhibited by caspase-1 and caspase-3 inhibitors

**Fig 5:**
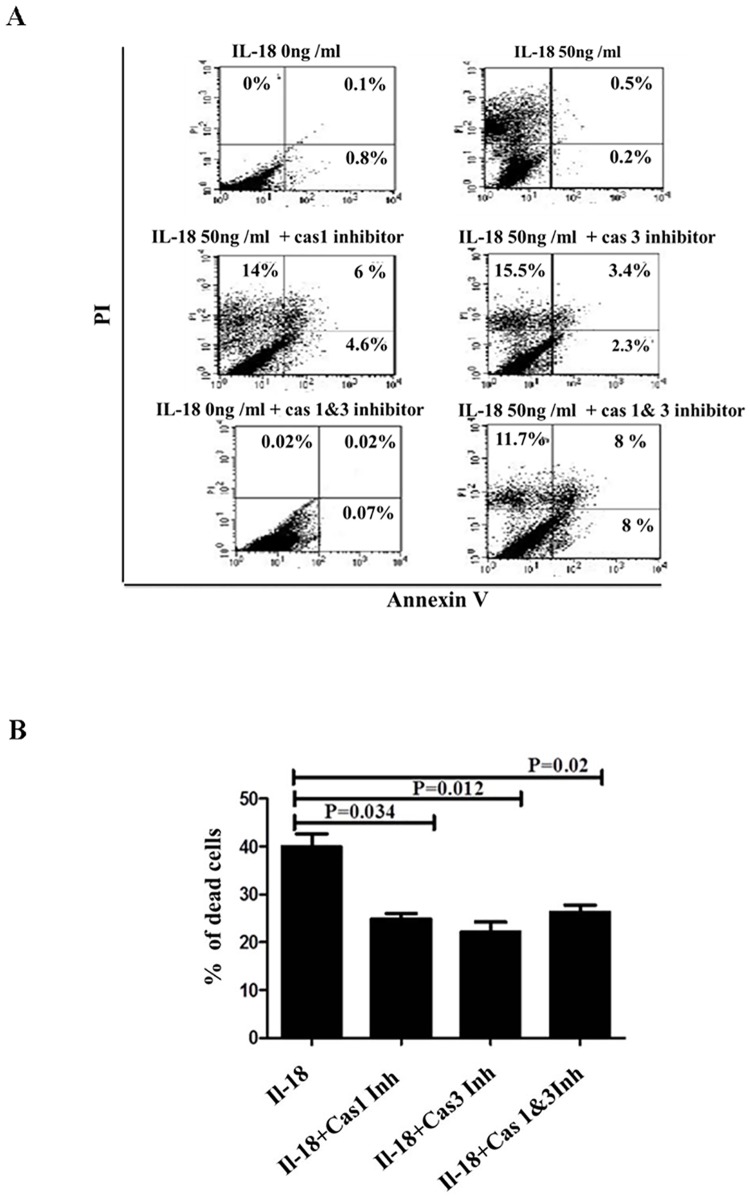
IL-18-induced cell death is partially inhibited by inhibitors of caspase-1 and caspase-3. HT29 cell monolayers were pre-treated with cell permeable inhibitors for caspase-1 (Z-YVAD-FMK), caspase-3 (Z-DQMD-FMK) or both (50 uM each) for 30 minutes. After 24 hours, the cells were collected and apoptosis was determined by staining with FITC-annexin V and PI. Panel **A** shows dot plots of the cells stained for Annexin V and PI. Panel **B** summarizes the results from three independent experiments by showing mean %ages ± SE.

Since capase-1 and capase-3 can induce cell death, we sought to determine the caspase involved in the IL-18-induced cell death. For this purpose, the cells were incubated with the cytokine (50 ng per ml) with and without the presence of cell permeable inhibitors specific for one or the other caspase or together (50 μM). The inhibitors were added to the cells 30 minutes prior to addition of the cytokine. As shown in the Fig 5 (panels A and B), each of the caspase inhibitors partially inhibited death in HT-29 cells. Together, the two inhibitors had no added effect. These results suggest that both caspase-1 and caspase-3 are implicated in the same mechanism of cell death.

### IL-18 and LPS both activate caspase-1 and caspase-3 in HT-29 cells

**Fig 6:**
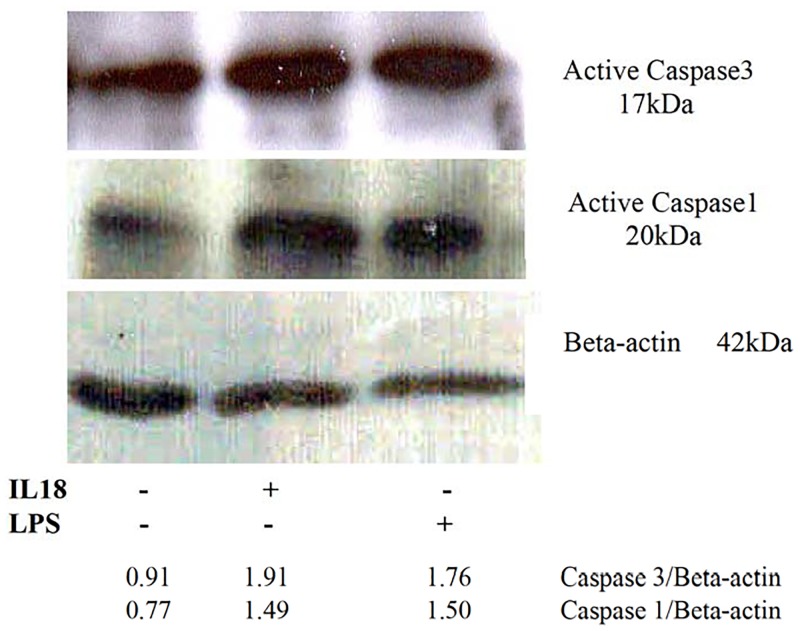
IL-18 and LPS activate caspase-1 and caspase-3 in HT-29 cells. The cell monolayers were treated with IL-18 (10 ng/ml) or with LPS (10 ng/ml) for 12 hours. Thereafter, the monolayers were washed with PBS, lysed and the activation of the caspases was determined on Western blots by using antibodies specific to the activated forms of the caspases. The Figure shows results from a representative of three independent experiments.

As previous experiment suggested the involvement of caspase-1 and caspase-3 in the cytokine-induced death of HT-29 cells, we sought to determine whether the cytokine activates these caspases. For this purposes, the cells were treated with IL-18 (10 ng per ml) or LPS (10 ng per ml). LPS is known to activate both caspases and was used as a positive control [41]. The activation of the caspases was determined by Western blots using antibodies specific for activated caspase-1 and activated caspase-3. As shown in Fig 6, LPS and IL-18 each activated the two caspases in HT-29 cells.

### IL-18 causes disruption and redistribution of β-catenin in HT-29 monolayers

**Fig 7:**
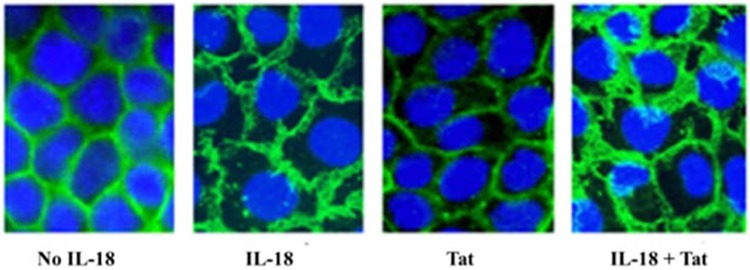
IL-18 disrupts and redistributes β-catenin in adherent junctions in HT-29 monolayers. The HT-29 monolayers were treated with IL-18 (10 ng/ml), Tat (100 ng/ml) or both. After 24 hours, the monolayers were incubated with rabbit anti-human β-Catenin or a control rabbit IgG (obtained from non-immunized animals as a negative control). After washing, the cells were stained with FITC-conjugated goat anti-rabbit antibodies, counterstained with DAPI, washed and examined under a fluorescent microscope. The figure shows typical images from three independent experiments. Note displacement and redistribution of the molecule around individual cells in the monolayers.

Since β-catenin plays an essential role in maintaining the integrity of adherens junctions by linking E-cadherin with cytoskeleton in epithelial cell monolayers [42], we sought to determine the effects of IL-18 on the distribution of β-catenin in the cell monolayers. We used Tat as a positive control, as it is known to disrupt these junctions in retinal pigmented epithelial cells [43]. As shown in Fig 7, IL-18 causes an increased expression but abnormal distribution of this molecule in the epithelial cell monolayers. In contrast, Tat significantly reduces its expression. When added together, the effects of IL-18 seem to predominate over those of Tat.

### IL-18 adversely affects expression of Tight Junction proteins in intestinal epithelial cell monolayers

**Fig 8:**
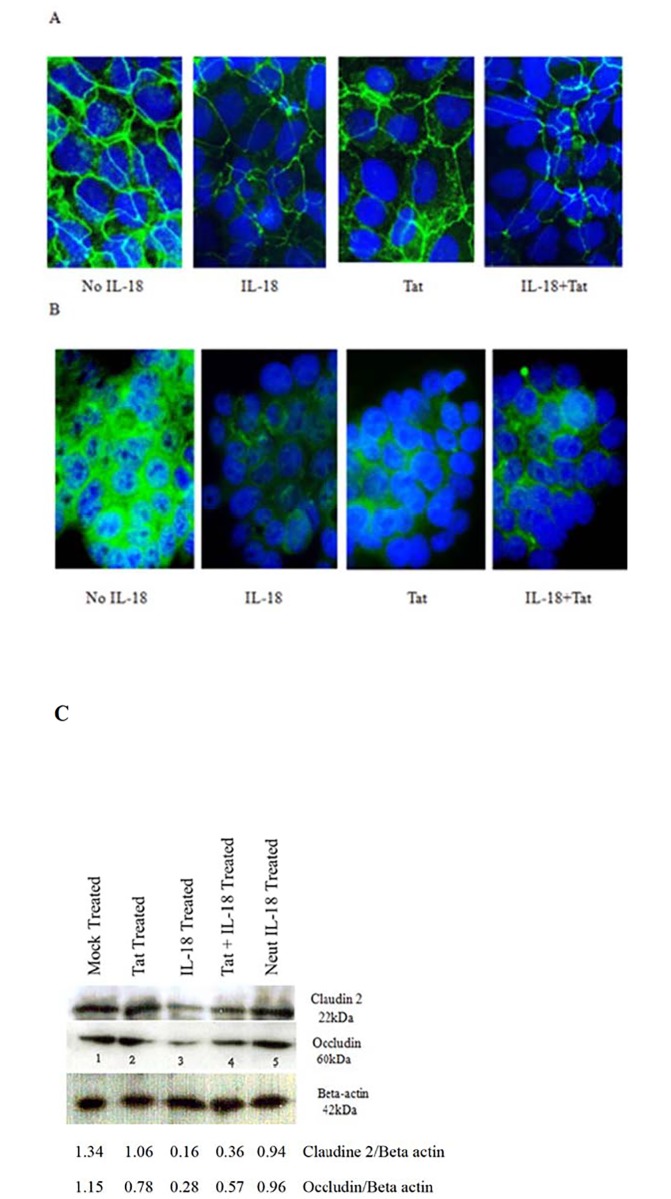
The effects of IL-18 on the expression of Tight Junction proteins in the intestinal cell monolayers. The cell monolayers were treated with IL-18 (10 ng/ml) and/or with Tat (100 ng/ml) for 24 hours, after which the cell monolayers were washed with PBS and stained with either protein specific rabbit antibodies or control antibodies (negative control), washed and stained with FITC-conjugated goat anti-rabbit antibodies. The cells were also stained with DAPI and examined under a fluorescent microscope and imaged. Staining of Caco2 monolayers for Claudin-2 (**A**) and of HT-29 monolayers for Occludin (**B**). The treatment of HT-29 monolayers also reduced expression of both Claudin-2 and Occludin when determined by Western blots (**C**).

Tight junctions are the main structures that regulate paracellular passage of biomolecules across epithelial cell monolayers [27]. In this regard, occludin and claudin-2 are important proteins in these junctions. Therefore, we sought to determine the impact of IL-18 on these proteins in the IEC monolayers. As shown in Fig 8 panel A, the cytokine (10 ng per ml for 24 hours) reduced expression of claudin 2 in the Caco-2 monolayers. The Tat treatment (100 ng per ml) also reduced the expression but less as compared with IL-18. As shown in Fig 8 panel B, the cytokine and Tat treatments also reduced expression of occludin in HT-29 monolayers. The treatment of HT-29 monolayers with the cytokine also reduced expression of both these proteins when determined by Western blots (Fig 8 panel C).

### IL18 decreases the expression of F-actin and disrupts its normal distribution around the cell cytoplasm

**Fig 9:**
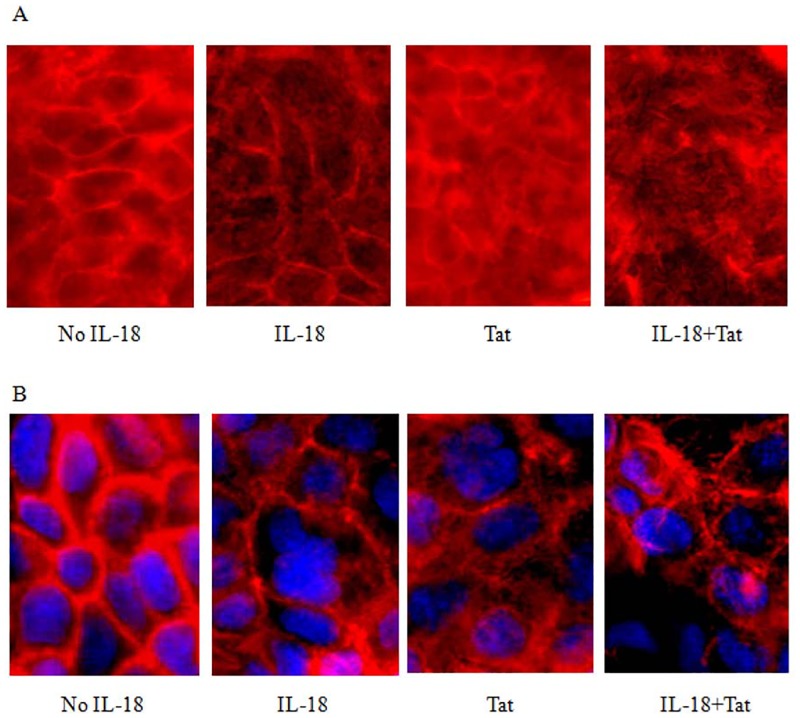
Effects of IL-18 on the expression and distribution of F-actin. The cells HT29 (**A**) and Caco2 (**B**) monolayers were treated with IL-18 and/or with Tat for 24 hours. The cells were washed with PBS, permeabilized, stained with Phalloidin red and DAPI. The cells were examined under a fluorescent microscope and photographed. The treatment with IL-18 causes a reduction in the expression whereas the treatment with Tat causes a more diffused expression of F-actin.

F- (or filamentous) actin is important for maintaining cell shape and motility in epithelial cells as well as for regulating intestinal permeability [44]. Therefore, we investigated the impact of IL-18 treatment (with and without Tat). As shown in Fig 9, both IL-18 and Tat cause a reduction and abnormalities in the expression of this cytoskeletal protein in both cell types. The effect was more clear and pronounced in HT-29 cells than in Caco2.

### IL-18 increases permeability in the intestinal epithelial cell monolayers

**Fig 10:**
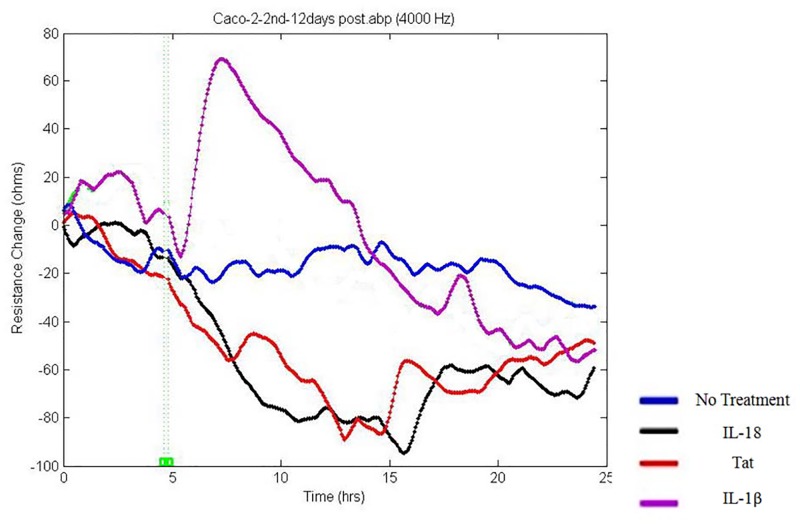
IL-18 decreases TEER in Caco2 monolayers. The TEER was measured during 24 hours after addition of 10 ng/ml of IL-18, 10 ng/ml IL-1β or 100 ng/ml Tat to the cell monolayers. Note dramatic decrease of the TEER occurring at hour 6 post-exposure by both IL-18 and Tat. IL-1β causes a transient increase in the TEER followed by a decrease beginning at hour 14 post-exposure. TEER was measured by the Electric Cell-substrate Impedance Sensing ECIS Zθ instrument and 8W10E+ electrode arrays were used.

As IL-18 causes several changes in cell shape and in the expression of cytoskeletal and Tight Junction proteins, the cytokine may adversely affect para-cellular permeability in the intestinal epithelial cell monolayers. Therefore, we sought to investigate the impact of this cytokine on this parameter in Caco2 cell monolayers by measuring trans-epithelial-monolayer electrical resistance (TEER). The TEER was measured using an electrical cell-substrate impedance sensing system (Applied Biophysics, Troy, USA) as described in the Materials & Methods section. As shown in Fig 10, the cytokine induced a decrease in the TEER that began at hour 5–6 post-exposure in the case of IL-18. The decrease persisted until 24 hour post-exposure. Interestingly, Tat and IL-18 had a similar effect on TEER in the cell monolayers.

### IL-18 affects permeability when applied from apical but not from basolateral surface of the intestinal epithelial cells

**Fig 11:**
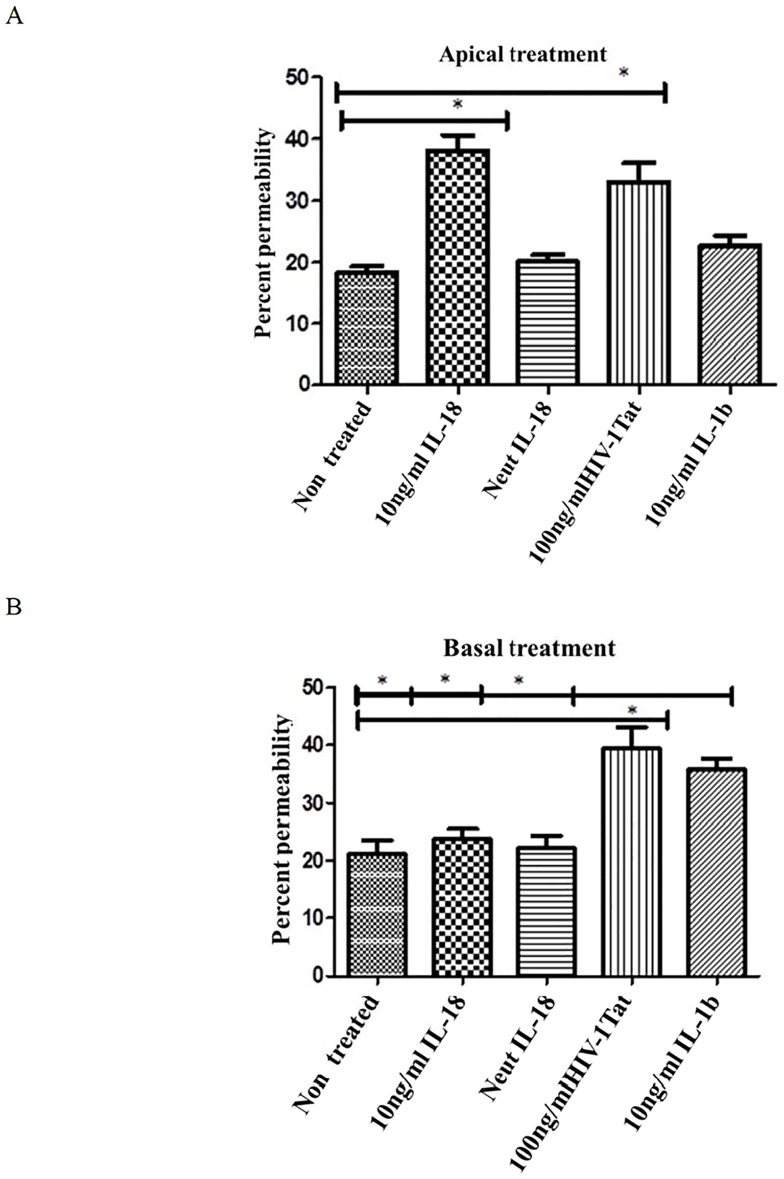
IL-18 increases paracellular permeability when applied to the apical surface. HT-29 cells were grown in monolayers in Transwell chambers. When confluent, IL-18 (10 ng /ml), IL-1β (10 ng/ml) or Tat (100 ng/ml) were applied to the apical surface **(A**) or to the Transwell bottom wells (**B**). In one well, we added IL-18 pre-neutralized with anti-IL-18 antibody. After 24 hours’ exposure, Lucifer Yellow (0.45 kDa; 100 nM) was added to the top wells. After 30 minutes, the Transwell upper chambers were removed, and the concentrations of the fluorescent tracer were measured in bottom wells in an ELISA reader. Percent permeability was determined by comparing the tracer in the treated bottom wells with the bottom well to which the tracer was added (100 nM). Bars and vertical lines indicate means ± SE; (* indicates that P<0.05).

It has been shown that cytokines’ effects on para-cellular permeability in epithelial cell monolayers may depend upon whether they are applied to the apical or to the basolateral surface [45,46]. Therefore, we sought to determine whether application of IL-18 to the apical or basolateral surface of the HT-29 monolayer affected its ability to increase the permeability. To this end, we grew the cells in Transwells. When confluent, we added the cytokine either to the apical surface or the bottom well. After 24 hours’ exposure, we added the Lucifer Yellow tracer to top wells, and measured its concentrations in the bottom wells 30 minutes later. As shown in Fig 11 panel A, IL-18 increased permeability when applied to the apical surface. Neutralization of the cytokine with the cytokine neutralizing antibodies abrogated this effect. The cytokine, however, had no effect on the monolayer permeability when it was applied from the baso-lateral surface (Fig 11 panel B). Contrary to this, IL-1β exerted its effect on permeability from the baso-lateral, but not from the apical, application. Interestingly, Tat increased the monolayer permeability from both the surfaces.

### IL-18 increases expression of MLCK and induces phosphorylation of MLC via ROCK

**Fig 12:**
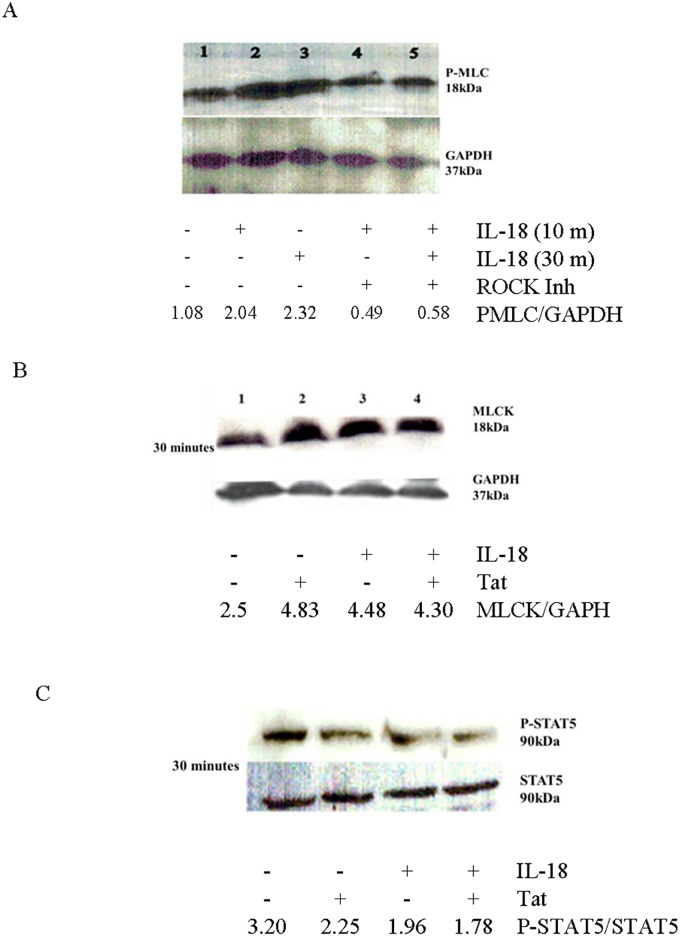
IL-18 increases expression of MLCK and pMLC via ROCK, and decreases that of phosphorylated STAT-5. HT29 cell monolayers were treated with IL-18 (10 ng/ml) and/or Tat (100 ng/ml) with or without a cell permeable inhibitor of ROCK (GSK429286A, 10μM; added 30 minutes prior to the addition of the cytokine). At the indicated time points, the cells were lysed. About 25 ug of the lysate proteins were resolved on SDS-PAGE and the Western blots were performed. Panel **A** shows results of a representative of three experiments for the expression of p-MLC. Panel **B** shows expression of MLCK after treatment of the monolayers with IL-18 (10 ng per ml) and/or with Tat (100 ng/ml) after 30 minutes. Panel **C** shows expression of pSTAT-5 and STAT-5 from IL-18 (10 ng per ml) and/or with Tat (100 ng per ml)-treated cells.

Some other cytokines (e.g., TNF-α and IL-1β) have been shown to disrupt intestinal integrity by increasing expression of MLCK and inducing phosphorylation of MLC by a pathway that involves ROCK [47]. Therefore, we were interested in determining whether IL-18 also follows the same pathway for disrupting integrity of HT-29 monolayers. For this purpose, we treated the cell monolayers with IL-18 (10 ng per ml) with and without a cell-permeable ROCK inhibitor (GSK429286A, 10μM) and determined the expression of pMLC at 10 and 30 minutes after the treatment. The inhibitor was added to the cell cultures 30 minutes before addition of the cytokine. As shown in Fig 12 panel A, the cytokine treatment increased expression of pMLC at both 10 and 30 minutes after the treatment. The increase was maximal at 10 minutes after the exposure. Furthermore, pre-treatment of the cells with a cell-permeable ROCK inhibitor inhibited the cytokine-induced increased in the expression of p-MLC (Fig 12 panel A). We also investigated whether the increased expression of p-MLC was accompanied with an increased expression of MLCK. As shown in Fig 12 panel B, IL-18 also induced a prominent increase in the expression of MLCK. Since STAT5 is known to promote intestinal wound healing and antagonize MLCK-mediated loss of intestinal barrier function [48], we sought to investigate the effects of IL-18 treatment on activated STAT5 in these IEC. Both IL-18 and Tat tended to reduce pSTAT-5 in the cell monolayers when they were examined 30 minutes after the treatments (Fig 12 panel C). Collectively, these data suggest that IL-18 uses the same signaling pathway for disrupting integrity of intestinal epithelial cell monolayers that are used by TNF-α and IL-1β [49,50].

### Serum concentrations of IL-18 and LPS correlate with each other in HIV-infected individuals and healthy controls

**Fig 13:**
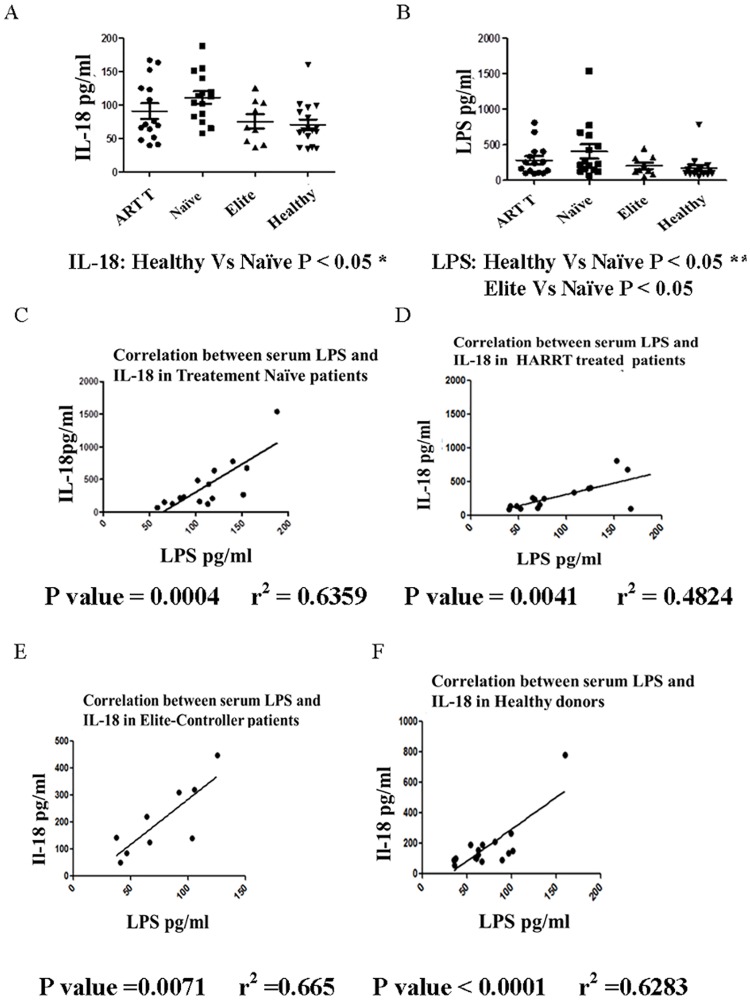
Correlation between serum IL-18 and LPS concentrations in HIV-infected and healthy control subjects. Concentrations of serum IL-18 **(A**) and LPS (**B**) in different groups of HIV-infected individuals. The vertical lines show medians ± SD. The other panels show correlation between the two parameters in treatment-naïve HIV-infected (**C**), HAART-treated HIV-infected (**D**), Elite-Controller (**E**), and HIV-seronegative healthy control individuals (**F**).

As we determined that IL-18 was a determinant of intestinal permeability, and microbial translocation has been shown to be a major factor in aberrant immune activation and AIDS pathogenesis [51,52], we sought to determine whether serum concentrations of the cytokine correlated with those of LPS in the serum. To investigate this, we measured IL-18 in the sera of HIV-infected patients belonging to different categories i.e., treatment-naïve HIV-infected, HAART-treated HIV-infected, Elite controllers and HIV-seronegative healthy control subjects. The treatment naïve HIV-infected patients had significantly higher concentrations of IL-18 and LPS compared with the control subjects (p<0.05); Fig 13 panel A and B. In these respects, the Elite controllers were not different from the healthy individuals. More importantly, in all the donor groups including the healthy control subjects, significant correlations were observed between IL-18 concentrations and LPS levels (Fig 13 panels C-F). These data strongly suggest IL-18 as a major factor in increasing intestinal permeability and causing microbial translocation in HIV-infected individuals as well as in healthy subjects.

## Discussion

We show here for the first time that HIV increases expression of mature IL-18 in a human intestinal epithelial cell line. However, the virus decreases the expression of IL-18BP, a naturally occurring antagonist for IL-18. We also found that the viral treatment increases concentrations of mature IL-18 but reduces those of IL-18BP in the culture media of these cells. Previous studies from our [17,18] and other laboratories [19,20,53] have shown that concentrations of IL-18 are increased in the circulation of HIV-infected individuals but those of its antagonist, IL-18BP, are either decreased or are not correspondingly increased. This imbalance in the production of IL-18 and IL-18BP results in increased amounts of free, biologically active IL-18 in the circulation of HIV-infected individuals. Treatment of these patients with anti-retroviral drugs reduces these levels but they tend to remain higher than their normal levels seen in HIV-seronegative healthy individuals; reviewed in [2, 5]. Sustained higher levels of the cytokine have been shown to serve as a surrogate marker for treatment failure in HIV-infected individuals [53].

IL-18 is produced from a wide variety of human cells including monocytes, macrophages, dendritic cells, intestinal epithelial cells as well as from the adrenal cortex [3,54,55]. However, the production of its antagonist, IL-18BP, in the body is more ubiquitous. The antagonist is produced as a negative feedback in response to a rise in the levels of IL-18, either locally or systemically. Most of IL-18 in the circulation is bound with IL-18BP and is inactive. An increase in its production also results in increased production of IL-18BP. IL-18 does so by inducing IFN-γ from NK, T and other cell types in the body. Production of IFN-γ is essential for transcriptional activation of the gene for IL-18BP, as IFN-γ KO mice have little detectable IL-18BP in their circulation [56]. Previously, we have shown that both T- and M-tropic viruses increase expression of IL-18 and decrease that of IL-18BP from human monocyte-derived macrophages [18]. Our present results suggest that the virus exerts similar effects on intestinal epithelial cells. In this connection, platelets may also contribute to this imbalance. We showed previously that IL-18 concentrations in the circulation of HIV-infected individuals correlate with the degree of platelets activation [57]. Moreover, we also showed that human platelets, upon activation, synthesize and release IL-18 but only release pre-made IL-18BP. Furthermore, HIV-1 interacts with these anucleate cells and induces expression of IL-18 but reduces that of IL-18BP [5]. Thus, the virus induces imbalance in the production of IL-18 and its antagonist by interacting with more than one cell type. Furthermore, we suggest that adrenal cortex may also contribute towards this imbalance. HIV-infected individuals are likely to experience increased psychosocial stress, which is known to activate the Hypothalamus-Pituitary-Adrenal (HPA) axis resulting in increased IL-18 secretion from adrenal cortex [58].

The imbalance in the production of IL-18 and its antagonist inevitably results in increased biological activities of the cytokine, which may have tissue destructive effects. The cytokine has been shown to exert death in several human cell types. It was shown to cause death in human cardiac vascular and umbilical vein endothelial cells [9,40,59]. We have previously shown that recombinant human IL-18 induces FasL expression in human NK cells. The IL-18-treated NK cells exert fratricidal effects via Fas-FasL interactions [8]. Furthermore, chronic exposure to IL-18 renders NK cells immunoablative by inducing expression of PDL-1 on them [60]. Within the gut, IEC-secreted IL-18 was recently shown to inhibit differentiation of TH17 cells and promote expansion of FoxP3+ regulatory T cells (Treg) [61]. It may be relevant to mention here that increased Treg/TH17 ratios, observed in the gut of HIV-infected individuals, are believed to play a role in increased intestinal permeability and microbial translocation [62]. As HIV replicates intensively in the gut-associated lymphoid tissues, and the virus induces this cytokine from the intestinal epithelial cells, we sought to determine its potential effects on these cell monolayers. The cytokine decreased trans-epithelial electrical resistance (TEER) in fully confluent intestinal epithelial cell monolayers. The IEC line, Caco2, used in these assays, has been extensively used for this purpose [63]. It is noteworthy that TEER is an indirect measure of the paracellular permeability of the cells grown in monolayers [64,65]. These results suggest that IL-18 increases paracellular permeability in these cell monolayers. Our results on the effects of IL-1β on the intestinal permeability as measured by TEER (an early increase followed by a decrease) are in agreement with earlier studies [66].

We also measured permeability of the fully confluent IEC monolayers by using a fluorescent tracer (Lucifer Yellow). Being 0.45 kDa in size, the tracer crosses the intestinal cell monolayer only if the Tight Junctions permeability is increased. Interestingly, IL-18 increased permeability of the HT-29 monolayers when it was applied to the apical surface of the monolayer but not when it was applied to its basolateral surface. On the contrary, IL-1β, which is the prototype member of the IL-1 family, increases the permeability from basolateral surfaces but not from apical surfaces. The cytokine has been shown earlier to increase intestinal permeability when applied from the basolateral surface to intestinal cell monolayers [46,50]. To the best of our knowledge, no study has previously investigated its effects when it is applied to the monolayers from their apical surface. Our results strongly suggest that the receptor for IL-1β is expressed on basolateral surface of the IEC, whereas that of IL-18 is restricted to apical surface of these cells. Further studies are required to confirm these findings. Our results are in agreement with a recent report that demonstrated that ICE-produced IL-18 contributes towards defective intestinal barrier function and inflammation in DSS-induced colitis in mice [12]. The difference between Tat and IL-18 on intestinal permeability, when applied to apical and basolateral borders, suggests that this viral protein may be acting more than by just inducing the secretion of IL-18 in the IEC.

We report here for the first time that HIV and its protein Tat induce IL-18 from intestinal epithelial cell line, HT-29. The cytokine increases intestinal permeability by disrupting intercellular junctions and by inducing apoptosis of the IEC. The cytokine induces increased expression of MLCK, which phosphorylates MLC. The phosphorylation of MLC is essential to cause contraction of the actomyosin cytoskeletal ring and consequently an increase in the Tight Junction permeability [67]. We also noted significant changes in the expression and distribution of some Tight Junction (occludin and claudin 2) and the Junctions Adherens protein (β-catenin). Claudin-2 is paracellular pore-forming protein and its increased expression correlates with increased permeability of the Tight Junctions [68]. Microbial products often increase its expression to overcome intestinal barrier [69]. IL-6, an important pro-inflammatory cytokine, has been shown to increase expression of claudin-2 in intestinal epithelium [70]. A decreased expression of this protein upon IL-18 treatment was unexpected. It may be due to prolonged incubation of IEC with the cytokine. Studies with TNF-α have shown that the cytokine increases expression of claudin-2 in IEC upon short term (1–6 hour) exposure but decreases the expression of this TJ protein upon longer exposures [70]. Similar to IL-18, IL-1β also reduces expression of occludin in corneal epithelial cells [71]. Pro-inflammatory cytokines such as TNF-α, and IFN-γ decrease expression of occludin by promoting its endocytosis and degradation in lysosomes. A reduced expression of occludin increases permeability of Tight Junctions. IL-18, like TNF-α and IFN-γ, increases expression of MLCK, which induces phosphorylation of MLC and increases intestinal permeability [72]. Collectively, these results suggest that IL-18 uses multiple mechanisms to increase intestinal permeability.

As mentioned, activated STAT-5 has been shown to antagonize MLCK-mediated loss of intestinal barrier function and promote intestinal wound healing [48]. In this regard, IL-18 has been previously shown to inhibit/reduce STAT-5 activation in different cell types other than IEC [73]. Therefore, we sought to investigate the effects of this cytokine on the activation of this transcription factor in these IEC. When examined 30 minutes post exposure (a time point that corresponds to increased expression of p-MLC in IL-18-treated IEC, the cytokine inhibited the activation of STAT-5. The inhibition of activated STAT-5 may play a role in reduced viability of the IEC and increased permeability of the IEC monolayers. It is well known that increased intestinal permeability results in increased translocation of bacterial products, fragments and even whole bacteria into body tissues and general circulation [74]. The microbial products like LPS cause a generalized activation of the immune system observed in HIV-infected individuals [26]. We also investigated concentrations of LPS (a proxy for microbial translocation) and IL-18 in the circulation of HIV-infected and HIV-seronegative healthy individuals. Interestingly, we found significant positive correlations between these two parameters in different HIV-infected individuals as well as in healthy donors. We are not aware of any other pro-inflammatory cytokine which has been reported to correlate with the concentrations of the circulating LPS. These results suggest that IL-18 plays a fundamental role in enhancing paracellular permeability and inducing microbial translocation in intestinal epithelial cell monolayers.

We observed that addition of IL-18 to the intestinal epithelial cell monolayers caused significant changes in their morphology. The cells became rounded, died and started floating in the culture medium. We observed that the cells underwent apoptosis, as they first became positive for FITC-Annexin V (a marker for early apoptosis) and then for a vital dye, PI (a marker for late apoptosis when cells lose integrity of their membranes). The cytokine also induced activation of caspase-1 and caspase-3 in the intestinal epithelial cells. Increased apoptosis and turn-over of enterocytes occurs in HIV-induced enteropathy [75]. Consequently, the villi become atrophied [76]. Our results suggest that the virus-induced IL-18 plays an important role in the enteropathy observed in HIV-infected individuals.

As mentioned above, increased concentrations of IL-18 are observed in the circulation of HIV-infected individuals [2,17–20,53]. Highly active anti-retroviral therapy (HAART) is well known to effectively suppress viral replication below detectable limits. It also reduces IL-18 levels in the circulation of these patients. The levels, however, remain above their normal values due to the persistence of low grade viral replication in HIV-infected patients despite undergoing HAART [2,77,78]. Low grade replication of the residual virus in anatomical viral sanctuaries such as gut is the main driver of this inflammation. At least in part, this may occur due to increased production of IL-18 without a corresponding increase in IL-18BP from IEC and myeloid cells. Increased production of this and other pro-inflammatory cytokines acts as a positive feed-forward loop to drive HIV replication, defective intestinal barrier, microbial translocation and chronic low-grade inflammation [12,21,22]. The low grade chronic inflammation puts HIV-infected individuals at risk for immunosenescence, premature aging, frailty, metabolic syndrome, cardiovascular diseases, dementia, cancer and HIV-associated lipodystrophy (HALS) characterized by the disappearance of sub-cutaneous fat (“empty cheek syndrome”) and its accumulation at unusual anatomical sites like the back of neck (“buffalo hump”), etc [79–81]. It is noteworthy that IL-18 inhibits differentiation of pre-adipocytes into adipocytes and promotes lipolysis [82]. Increased expression of the cytokine was observed at the sites of depletion of subcutaneous fat in HIV-infected individuals [81,83]. Increased concentrations of the cytokine are a risk factor for heart attack, type 2 diabetes and dementia [84,85]. Today, HIV-infected individuals on anti-retroviral therapy rarely die due to AIDS, but suffer from chronic low-grade inflammation that leads to several clinical conditions that are not associated with AIDS. Increased concentrations of IL-18 may play a crucial role in inducing these conditions and its neutralization should be considered as a therapeutic option.

We used Tat in some experiments to determine its effects on intestinal epithelial cells. Tat is a versatile protein that has been shown to bind TLR4-MD2-CD14 complex, activate NF-κB pathway and induce pro-inflammatory cytokines [86,87]. It also induces apoptosis in enterocytes and plays an important role in the pathogenesis of HIV-induced AIDS [88,89]. We found that the tansactivator also exerts similar effects on the IEC concerning production of IL-18 and its antagonist. However, subtle differences were observed between IL-18 and Tat on the expression of different intestinal junction proteins. We show here for the first time that it induces production of IL-18 from a human IEC line. The induction may represent another aspect of Tat’s multiple effects on human cells.

In summary, our results show that HIV exerts differential effects on the production of IL-18 and its antagonist from intestinal epithelial cells. The virus induces activation of caspase-1 resulting in increased processing of pre-cursor IL-18 and the release of mature IL-18 into the culture media. The cytokine increases intestinal permeability and translocation of microbial products into systemic circulation. It also promotes apoptosis of intestinal epithelial cells. The caveat is that all these results were obtained in established human intestinal epithelial cells (IEC) and need to be confirmed in primary human IEC. If confirmed, they would suggest that IL-18 plays an important role in HIV-induced enteropathy and should be considered a target molecule for reducing HIV-induced pathology.
